# Refining Glaucoma Management: Korean Population-Specific Findings from Visual Field and Optical Coherence Tomography Testing

**DOI:** 10.3390/biomedicines13020318

**Published:** 2025-01-29

**Authors:** Sun Jung Lee, Sung Hun Jang, Jae Kyung Kim

**Affiliations:** 1 Department of Biomedical Laboratory Science, College of Health and Welfare, Dankook University, Cheonan-si 31116, Republic of Korea; sunj0509@gmail.com; 2 Department of Medical Laser, Graduate School of Medicine, Dankook University, Cheonan-si 31116, Republic of Korea; well8143@naver.com

**Keywords:** ganglion cell–inner plexiform layer, Korea, optical coherence test, retinal nerve fiber layer, visual field test

## Abstract

Background/Objectives: This study aimed to serve as a reference for establishing glaucoma evaluation standards for the Korean by analyzing the results of visual field tests and optical coherence tomography (OCT). We also determined the correlation between these test results and patient demographics, such as age and sex, which is crucial for early glaucoma detection and management. Methods: This study was conducted at a national hospital in Seoul and analyzed 1510 visual field tests and 1337 OCT tests. The patients underwent the Humphrey automated visual field test and OCT measurements. Glaucoma was classified into early, moderate, and advanced stages based on the mean deviation (MD) value. Statistical analyses were performed to assess the relationships between age, sex, and test results. Results: The visual field test results showed that the visual field index and MD values decreased as age increased for both males and females, with a more significant decrease observed in males. The OCT findings revealed gradual thinning of the ganglion cell–inner plexiform layer (GCIPL) and retinal nerve fiber layer (RNFL) with increasing age, although, compared to males, thicker GCIPLs and RNFLs were maintained in females until their 60s. Conclusion: This study indicates that there may be an association between age and sex in glaucoma progression and provides valuable insights for establishing diagnostic and management modalities specifically for Korean patients. These findings support the use of both visual field testing and OCT in glaucoma diagnosis, emphasizing the importance of early detection and personalized treatment strategies for effective glaucoma management in Korea.

## 1. Introduction

Glaucoma refers to a collection of conditions characterized by the gradual deterioration of the optic nerve, leading to the loss of retinal ganglion cells, thinning of the retinal nerve fiber layer, and progressive enlargement of the optic disc’s cupping [1]. Glaucoma is a significant eye condition that poses a serious threat to vision health and is one of the leading causes of irreversible blindness worldwide [2]. Currently, it is estimated that over 60 million individuals around the world are affected by glaucoma, and this number is projected to increase dramatically to 112 million by 2040 [3]. This alarming increase emphasizes the need for enhanced diagnostic, preventive, and treatment strategies. Glaucoma, classified as a neurodegenerative disorder, is characterized by the progressive deterioration of retinal ganglion cells [4]. The optic nerve, which is essential for transmitting visual information from the retina to the brain, is progressively damaged, leading to gradual narrowing of the field of vision. If left untreated, this condition can result in significant visual impairment and eventual blindness [4]. Early detection and timely management of the disease are critical for preserving vision and maintaining quality of life.

Two primary diagnostic tools have been found to be indispensable for the detection and monitoring of glaucoma: visual field testing and optical coherence tomography (OCT) [5]. These tools play complementary roles in the identification of both the functional and structural changes associated with the disease. Glaucoma is a silent disease, often progressing without noticeable symptoms until significant damage has occurred. Peripheral vision loss, one of the earliest signs, can go unnoticed by patients, making regular screening and early intervention all the more critical. By combining the strengths of visual field testing and OCT, clinicians can achieve a more comprehensive understanding of the disease, enabling them to make informed decisions about diagnosis, staging, and treatment. The findings of each modality provide unique insights that are critical for assessing disease progression and formulating individualized treatment plans.

The visual field test is a cornerstone in the evaluation of functional vision loss caused by glaucoma [5]. This test is particularly effective in identifying visual field defects due to the disease [3]. During the procedure, the patient remains in a fixed position, while lights of varying brightness are projected onto specific areas of the patient’s visual field [6]. Sensitivity to these lights is then measured on the basis of the patient’s responses. This approach is especially valuable for the detection of changes in peripheral vision, which is the most vulnerable to glaucoma-related damage [4]. Notably, alterations in peripheral vision often precede changes in central vision. In the early stages of the disease, the visual field test can detect vision loss even before it impacts visual acuity [7]. This makes the test an essential tool not only for initial diagnosis, but also for ongoing monitoring of disease progression.

In contrast, OCT focuses on identifying structural changes in the retina and optic nerve [8]. This advanced imaging technology provides detailed, high-resolution images of the retinal nerve fiber layer (RNFL) and optic disc, enabling the early detection of nerve damage caused by glaucoma. Structural changes, such as thinning of the RNFL and alterations in the optic disc, often occur before functional deficits become apparent [9]. OCT allows for both qualitative and quantitative analysis of these parameters, making it a highly effective tool for tracking disease progression [8]. Furthermore, the thickness of the RNFL is considered a reliable indicator of early glaucomatous damage [8], underscoring the importance of OCT in early diagnosis and management.

Global studies have highlighted the utility of these diagnostic tools. For instance, research from a university in Auckland, New Zealand reported an average mean deviation (MD) of −5.91 ± 7.72 (SD) dB in visual field testing [10]. Meanwhile, a study from the Wilmer Eye Institute in Baltimore, USA documented baseline RNFL measurements of 83.6 mm and an MD of −1.75 dB in visual field tests for patients with glaucoma or suspected glaucoma [11]. These findings demonstrate the value of visual field and OCT tests for providing measurable indicators of glaucoma severity and progression.

However, despite the wealth of international research, studies focusing on the Korean population remain limited. This lack of data has posed challenges in the development of diagnostic and management guidelines tailored to the specific characteristics of Korean patients. Recognizing this gap, recent research has aimed to address the issue by analyzing data from patients undergoing visual field and OCT tests at a hospital in Seoul, Korea. The present study aimed to establish glaucoma evaluation standards for the Korean population by analyzing the results of visual field tests and OCT. We also determined the correlation between these test results and patient demographics, such as age and sex, which is crucial for early glaucoma detection and management.

The significance of this research lies in two key areas. First, by utilizing data specifically obtained from Korean patients, it provides insights that can inform the development of localized diagnostic and treatment criteria. This is particularly important, given the unique demographic and genetic factors that may influence the presentation and progression of glaucoma in this population. Second, the study highlights the importance of preventive measures for individuals in the early stages of the disease. By examining the correlation of visual field test and OCT findings with demographic factors, such as age and sex, the research underscores the need for personalized and proactive approaches to glaucoma management [12].

## 2. Materials and Methods

### 2.1. Ethics Approval

This study was approved by the Dankook University Institutional Review Board (IRB Approval Number: 2022-08-010) and was carried out following the principles outlined in the Declaration of Helsinki. Informed consent from the participants was not required because the study relied on data from diagnostic tests conducted by medical facilities and did not involve any personal patient information.

### 2.2. Participants

Patients who attended for glaucoma management and treatment between 30 May and 24 June 2022 at a national hospital in Seoul, South Korea, were randomly selected for this study. A total of 1510 visual field tests and 1337 OCT tests were reviewed.

Data obtained from visual field testing were studied on 639 men and 870 women, at rates of 42% and 58%, respectively. Additionally, data obtained from OCT examination were studied on 620 men and 717 women, at rates of 46% and 54%, respectively.

In most cases, both eyes of each patient were tested, and tests with results deemed inappropriate for analysis were excluded. Specifically, data with extremely low reliability, such as those obtained with decreased patient response, those obtained with decreased concentration during testing, or those with decreased reproducibility on repeated testing, were excluded from the analysis. Throughout this stage, the study focused only on reliable diagnostic data related to glaucoma mechanisms.

### 2.3. Visual Field Test

#### 2.3.1. Visual Field Test Strategy

The study incorporated both the full-threshold Swedish Interactive Threshold Algorithm standard and fast tests, with the majority of tests being of the former type. Only patients who underwent the Humphrey automated visual field test were included in this study.

#### 2.3.2. Types of Visual Field Tests

In general, visual field tests can be divided into three types (10-2, 24-2, and 30-2) based on the visual range to be checked. Among these, we obtained data from type 24-2 tests.

(1) 10-2: Evaluates the central 10°. Ideal for screening advanced glaucoma cases.
(2) 24-2: A pattern that reduces the temporal, superior, and inferior sides by 6°, except for the nose side in 30-2.
(3) 30-2: 30° range threshold assessment. This is the most frequently used threshold test.

#### 2.3.3. Classification of Glaucoma

(1) MD: Based on the MD value in the visual field test, glaucoma was categorized into early (−6 < MD < 0), moderate (−12 < MD < −6), and advanced (MD < −12) stages.

The MD value serves as a measure of overall alterations in the visual field. It is calculated by averaging the differences between the sensitivity (threshold) of the patient and the normal sensitivity (threshold) of age-matched individuals in a healthy population, with adjustments for age.

(2) Visual field index (VFI): VFI is a number ranging from 0 to 100 that quantifies the degree of damage to retinal ganglion cells. 100 indicates normal vision, and 0 indicates complete lack of vision, indicating the patient’s score. This value shows a linear correlation with the progression of glaucoma.

Therefore, VFI is helpful in checking whether glaucoma is progressing after glaucoma is diagnosed.

The *p* value for the average difference between the VFI and MD values for each age group is less than 0.05, which is significant. The *p* value for the mean difference for each sex is less than 0.05 for VFI and 0.08 for MD.

### 2.4. OCT

Patients who underwent OCT using the designated device were included in this study. Images of the optic nerve head were acquired using a 200 × 200 optic cube from the Cirrus HD OCT 6000, version 10.0 (Carl Zeiss Meditec, Dublin, CA, USA). Ganglion cell–inner plexiform layer (GCIPL) thickness measurements were obtained from a macular cube scan, centered on the macula, using a 200 × 200 pixel axial scan over a 6 × 6-mm region that included the macula and surrounding tissues. The optic disc cube scan was centered on the optic nerve head, employing an axial scan that covered a 6 × 6 mm region at a resolution of 200 × 200 pixels, capturing both the optic nerve head and adjacent areas.

The *p* value for the average difference between the GCIPL and RNFL values for each age group is less than 0.05, which is significant. The *p* value for the mean difference for each sex is 0.10 for GCIPL and less than 0.05 for RNFL.

### 2.5. Statistical Analysis

Data were analyzed using the MedCalc software package (20.218ver, MedCalc Software, Ostend, Belgium) after participants were matched according to age, sex, average RNFL and GCIPL thicknesses, and visual field test outcomes. Visual field data were categorized into stages based on MD reference values, and statistical analyses were conducted to explore the relationships between age and sex. Furthermore, correlations between GCIPL and RNFL thickness measurements and age and sex were determined.

## 3. Results

### 3.1. Analysis of Visual Field Test Results

**Figure 1:**
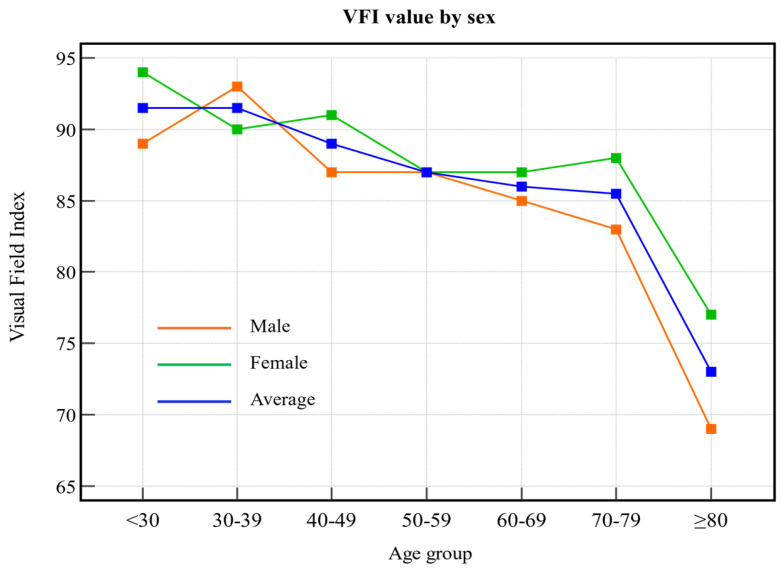
VFI values according to age group. The orange line indicates male sex, the green line indicates female sex, and the blue line indicates the average value. VFI: visual field index.

A total of 1510 visual field tests were reviewed. Analysis of graphs showing changes in the VFI by age showed that VFI values for males tended to be high in the younger age groups and gradually decreased with an increase in age (Figure 1). The VFI values for females remained relatively higher than those for males, although they also tended to decrease with increasing age. Overall, the mean VFI values tended to decrease with increasing age, indicating increasing damage to vision with advancing age.

The results of ANOVA showed significant differences in VFI and MD values among the eight age groups (*p* < 0.001 for both indicators). Post hoc Tukey’s Honestly Significant Difference (HSD) tests identified some significant pairwise differences. With regard to VFI values, the group aged ≥80 years showed a significantly lower value than did the group aged <30 years and the group aged 30 to 39 years (*p* < 0.05). With regard to MD values, significant differences were observed between the group aged ≥80 years, the group aged <30 years, and the group aged 30 to 39 years (*p* < 0.01).

**Figure 2:**
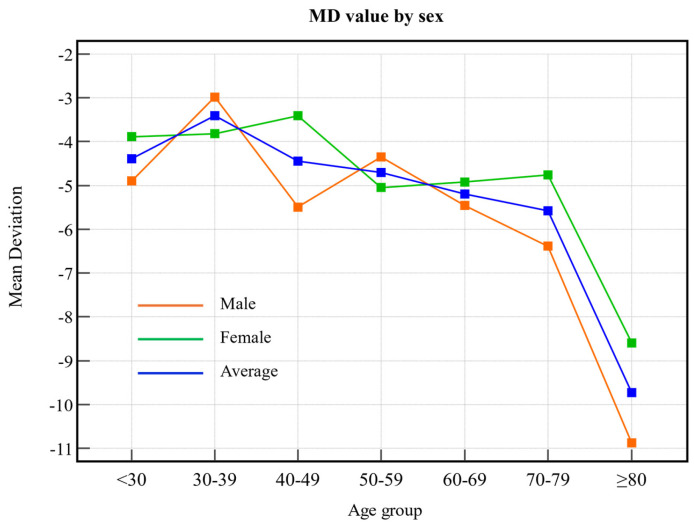
MD values according to age group. The orange line indicates male sex, the green line indicates female sex, and the blue line indicates the average value. MD: mean deviation.

MD values tended to decrease with increasing age for both males and females (Figure 2). In particular, the decrease was greater in males than in females. The MD value indicates the degree of visual field loss, with lower values indicating more severe visual field loss; thus, the findings of this study suggest that males may experience more severe visual field loss as they age.

**Figure 3:**
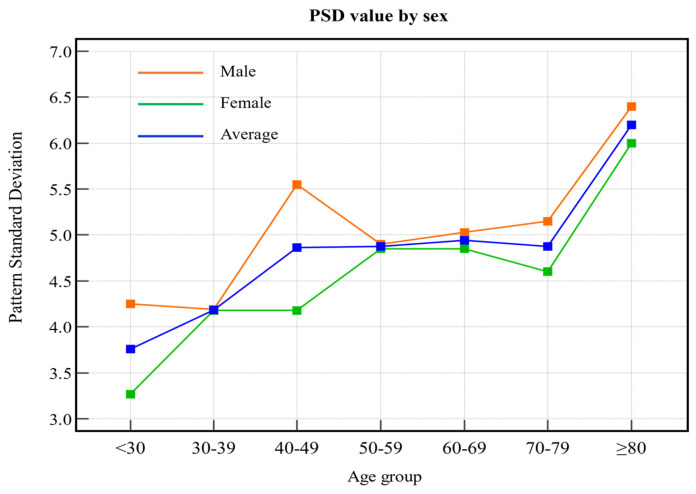
PSD values according to age group. The orange line indicates the male sex, green indicates the female sex, and blue indicates the average value. PSD: pattern standard deviation.

Analysis of the pattern SD (PSD) graphs revealed that PSD values fluctuated slightly with increasing age and tended to remain constant without significant changes (Figure 3). The PSD value indicates the nonuniformity of the visual field defect; the higher the value, the more nonuniform the visual field defect. Accordingly, the study findings suggest that the heterogeneity of visual field defects does not change significantly with age.

### 3.2. Analysis of OCT Findings

A total of 1337 OCT tests were reviewed. Analysis of GCIPL thickness values by age indicated that, for males, the values ranged from 78 to 64 µm and tended to decline with advancing age (Table 1). For females, GCIPL thickness values ranged from 67 µm to 61 µm and also demonstrated a declining trend with age, although there was a slight increase from the 50s to the early 60s. This suggests that ganglion cell layer thickness in females is relatively well preserved until a certain age.

The results of ANOVA revealed significant differences in RNFL and GCIPL values among the eight age groups (*p* < 0.001 for both indicators). Post hoc Tukey’s HSD tests identified some significant differences. With regard to RNFL values, compared with the thickness in the group aged <30 years and the group aged 30 to 39 years, significant thinning was observed in the group aged ≥80 years (*p* <0.05). With regard to GCIPL values, similar trends were observed, with significant differences observed between the group aged <30 years and the group aged ≥80 years (*p* <0.01).

Analysis of RNFL values by age indicated that, for males, these values decreased from 81 µm to 70 µm, suggesting a decline with advancing age (Table 2). For females, RNFL values decreased slightly from 75 µm to 71 µm and remained relatively stable from their 40s to their early 60s. This observation implies that RNFL thickness is comparatively well preserved in females up to a certain age.

## 4. Discussion

Glaucoma refers to a group of complex eye diseases that lead to gradual damage to the optic nerve, often resulting in progressive vision loss and, in severe cases, complete blindness [13]. This condition is widely recognized as one of the leading causes of irreversible blindness globally, and it imposes a significant public health burden. Factors contributing to an increased risk of glaucoma include advancing age, smoking, African descent, a family history of the disease, genetic predisposition, hypertension, hypotension (notably reduced nocturnal blood pressure), atherosclerosis, lipid imbalance, and diabetes [1]. These risk factors highlight the multifaceted nature of the disease and underline the importance of a comprehensive approach to its management. Early diagnosis and consistent monitoring are essential for preventing the progression of glaucoma. While glaucoma damage is irreversible, timely interventions can prevent further deterioration of vision [14].

As an irreversible disease, glaucoma necessitates strategies to detect visual field defects early and monitor the progression of visual field deterioration. This early detection is crucial because it significantly influences outcomes [15]. The current study, conducted in Korea, focused on patients undergoing glaucoma treatment at a hospital. It aimed to calculate average values obtained from visual field and OCT tests stratified by age and sex. The findings provide valuable insights into the impact of these demographic factors on glaucoma progression.

The visual field test evaluates three primary global metrics, VFI, MD, and PSD, to quantify functional changes in a patient’s vision [16]. VFI reflects the extent of damage to the retinal ganglion cells, which are critical for transmitting visual information. MD measures the overall deviation of a patient’s sensitivity from that of a healthy, age-matched population. PSD highlights localized changes in the visual field by quantifying deviations in its uniformity.

The inclusion of VFI alongside MD in modern devices, such as the Humphrey automated perimetry device, has simplified clinical applications. VFI expresses remaining the visual function as a percentage, making interpretation and monitoring easier [15]. Research indicates a strong correlation between VFI and MD, although the relationship becomes less distinct in cases of mild glaucoma [17].

Our study revealed age-related trends in these metrics. Both VFI and MD decreased as age advanced, with a sharp decline observed after 70 years. These findings suggest that older adults have more advanced glaucoma progression than younger adults, consistent with the understanding that glaucoma severity is strongly correlated with age. Our study showed that VFI and MD values gradually decreased with increasing age, and that the thicknesses of the GCIPL and RNFL also decreased. However, these changes are not limited to glaucoma. Normal aging is known to affect both the visual field and the optic nerve head; this suggests that some of the observed changes may reflect physiological aging rather than glaucoma progression. This highlights the need for sophisticated diagnostic criteria that account for the overlapping effects of aging and glaucoma. Conversely, PSD values increased with age, indicating an escalation in localized visual field defects. These trends were consistent across both sexes. Notably, while older adults may demonstrate slower reaction times and decreased accuracy during testing, visual field patterns remain detectable irrespective of age or sex [18].

In addition to visual field tests, OCT serves as a critical tool for identifying structural changes associated with glaucoma. OCT measures the GCIPL and RNFL thicknesses, both of which are key indicators of glaucomatous damage. These measurements are invaluable for detecting structural abnormalities before functional vision loss becomes apparent [19].

Compared to visual field testing, OCT is less time-consuming and less reliant on patient cooperation. This makes it particularly useful in clinical settings, where patient fatigue and limited testing capacities are challenges. Studies have demonstrated that OCT can detect glaucomatous changes up to six years before they manifest in visual field tests [19]. This underscores the importance of integrating both functional and structural assessments for a comprehensive evaluation of glaucoma progression.

In conclusion, glaucoma represents a growing global challenge that requires a multifaceted approach to detection, monitoring, and management. The integration of functional assessments through visual field testing and structural evaluations via OCT provides a powerful framework for addressing this complex disease. Continued research, particularly studies that focus on specific populations, will be essential for refining these approaches and ensuring that patients receive the most effective care possible.

In our analysis, GCIPL and RNFL thicknesses were found to decrease with age, with individuals in their 80s exhibiting significantly thinner layers than those in their 20s. Although this age-related decline was slightly more pronounced in males, the differences between the sexes were not statistically significant. Interestingly, the combined use of OCT and visual field testing improved diagnostic accuracy by over 17%, demonstrating the value of a multimodal approach [11].

While the findings of this study contribute valuable insights, several limitations must be acknowledged. The study’s short timeframe may limit the generalizability of the results. Additionally, as a retrospective study, it did not account for potential confounding factors, such as co-existing conditions like cataracts, which can influence test outcomes, particularly in elderly patients. In addition, intraocular pressure is an important clue in diagnosing glaucoma. Therefore, additional research is needed in addition to visual field testing and OCT testing. Future studies with larger, more diverse populations and extended observation periods are needed to validate these findings and refine diagnostic protocols.

Globally, glaucoma is the second leading cause of blindness, with its prevalence disproportionately affecting women and individuals of Asian descent [20]. Beyond its clinical implications, glaucoma significantly impacts health-related quality of life. Even in its early stages, vision impairment caused by the disease can disrupt daily activities, emotional well-being, and social participation. Timely diagnosis and proactive management are thus essential not only for preserving vision, but also for mitigating the broader personal, social, and economic burdens associated with glaucoma [21].

This study reaffirms that age is a significant factor in the progression of glaucoma, with older populations being more exposed to its effects. As a preventive measure, regular screening and testing should be prioritized for individuals as they age. Integrating demographic-specific insights, such as those derived from this research, into clinical practice can improve the effectiveness of glaucoma diagnosis and treatment, ultimately enhancing patient outcomes.

Glaucoma poses a complex challenge and requires a multifaceted approach to diagnosis, monitoring, and treatment. By combining the strengths of visual field testing and OCT, clinicians can gain a more nuanced understanding of both the functional and structural aspects of the disease. This comprehensive perspective is essential for tailoring interventions to individual patient needs and minimizing the impact of glaucoma on vision and quality of life.

## Figures and Tables

**Table 1:** GCIPL values according to age group.

	<30 years	30–39 years	40–49 years	50–59 years	60–69 years	70–79 years	≥80 years
Male	78 µm	67 µm	69 µm	70 µm	68 µm	65 µm	64 µm
Female	67 µm	68 µm	71 µm	67 µm	72 µm	69 µm	61 µm
*p* value	0.01	0.78	0.44	0.1	N/A	0.01	0.13

GCIPL: ganglion cell–inner plexiform layer, N/A: not applicable.

**Table 2:** RNFL values according to age group.

	<30 years	30–39 years	40–49 years	50–59 years	60–69 years	70–79 years	≥80 years
Male	81 µm	71 µm	74 µm	73 µm	72 µm	70 µm	70 µm
Female	75 µm	77 µm	77 µm	76 µm	77 µm	74 µm	71 µm
*p* value	0.24	0.08	0.12	0.09	N/A	N/A	0.76

RNFL: retinal nerve fiber layer, N/A: not applicable.

## Data Availability

The authors confirm that the data supporting the findings of this study are available within the article.

## References

[1] Schuster A.K., Erb C., Hoffmann E.M., Dietlein T., Pfeiffer N. (2020). The Diagnosis and Treatment of Glaucoma. Dtsch. Ärzteblatt Int..

[2] Tham Y.C., Li X., Wong T.Y., Quigley H.A., Aung T., Cheng C.Y. (2014). Global prevalence of glaucoma and projections of glaucoma burden through 2040: A systematic review and meta-analysis. Ophthalmology.

[3] Stapelfeldt J., Kucur S.S., Huber N., Höhn R., Sznitman R. (2021). Virtual reality-based and conventional visual field examination comparison in healthy and glaucoma patients. Transl. Vis. Sci. Technol..

[4] Weinreb R.N., Khaw P.T. (2004). Primary open-angle glaucoma. Lancet.

[5] Patel N.B., Sullivan-Mee M., Harwerth R.S. (2014). The relationship between retinal nerve fiber layer thickness and optic nerve head neuroretinal rim tissue in glaucoma. Investig. Ophthalmol. Vis. Sci..

[6] Bulbul E., Akar G.B. The effect of virtual reality and prediction in visual field test. Proceedings of the 30th Signal Processing and Communications Applications Conference (SIU).

[7] Lee J.W., Lee J.S. (2019). Visual field test. Primary Eye Examination: A Comprehensive Guide to Diagnosis.

[8] Chauhan B.C., Garway-Heath D.F., Goñi F.J., Rossetti L., Bengtsson B., Viswanathan A.C., Heijl A. (2008). Practical recommendations for measuring rates of visual field change in glaucoma. Br. J. Ophthalmol..

[9] Medeiros F.A., Zangwill L.M., Bowd C., Weinreb R.N. (2004). Comparison of the GDx VCC scanning laser polarimeter, HRT II confocal scanning laser ophthalmoscope, and Stratus OCT optical coherence tomograph for the detection of glaucoma. Arch. Ophthalmol..

[10] Kerr N.M., Chew S.S., Eady E.K., Gamble G.D., Danesh-Meyer H.V. (2010). Diagnostic accuracy of confrontation visual field tests. Neurology.

[11] Bradley C., Herbert P., Hou K., Unberath M., Ramulu P., Yohannan J. (2023). Comparing the accuracy of peripapillary OCT scans and visual fields to detect glaucoma worsening. Ophthalmology.

[12] Chen T.C. (2009). Spectral domain optical coherence tomography in glaucoma: Qualitative and quantitative analysis of the optic nerve head and retinal nerve fiber layer (an AOS thesis). Trans. Am. Ophthalmol. Soc..

[13] Wiggs J.L., Pasquale L.R. (2017). Genetics of glaucoma. Hum. Mol. Genet..

[14] Van Melkebeke L., Barbosa-Breda J., Huygens M., Stalmans I. (2018). Optical coherence tomography angiography in glaucoma: A review. Ophthalmic Res..

[15] Cho J.W., Nam Y.P., Kim D.Y., Kang S.Y., Sung K.R., Kook M.S. (2010). Clinical validation of visual field index. J. Korean Ophthalmol. Soc..

[16] Wang X., Chen H., Luo L., Ran A.R., Chan P.P., Tham C.C., Heng P.A. (2019). Unifying structure analysis and surrogate-driven function regression for glaucoma OCT image screening. Medical Image Computing and Computer Assisted Intervention—MICCAI 2019, Proceedings of the 22nd International Conference, Shenzhen, China, 13–17 October 2019.

[17] Sousa M.C., Biteli L.G., Dorairaj S., Maslin J.S., Leite M.T., Prata T.S. (2015). Suitability of the visual field index according to glaucoma severity. J. Curr. Glaucoma Pract..

[18] Koriat A., Norman J. (1985). Mental rotation and visual familiarity. Percept. Psychophys..

[19] Saheb H., Salimi A. (2022). Clinical applications of optical coherence tomography (OCT) in glaucoma. Can. Eye Care Today.

[20] Quigley H.A., Broman A.T. (2006). The number of people with glaucoma worldwide in 2010 and 2020. Br. J. Ophthalmol..

[21] Varma R., Lee P.P., Goldberg I., Kotak S. (2011). An assessment of the health and economic burdens of glaucoma. Am. J. Ophthalmol..

